# Juvenile Reactive Arthritis and other Spondyloarthritides of Childhood: A 28-year Experience from India

**DOI:** 10.31138/mjr.32.4.338

**Published:** 2021-12-27

**Authors:** Latika Gupta, R Naveen, Sakir Ahmed, Abhishek Zanwar, Durga P. Misra, Able Lawrence, Vikas Agarwal, Ramnath Misra, Amita Aggarwal

**Affiliations:** Department of Clinical Immunology and Rheumatology, Sanjay Gandhi Post Graduate Institute of Medical Sciences, Lucknow, India

**Keywords:** juvenile arthritis, reactive arthritis, spondyloarthritis, medical records review

## Abstract

**Objectives:**

Understanding of Juvenile reactive arthritis (jReA) and other spondyloarthritides of childhood (jSpA) is limited to small case series. Since most of them have speculated pathogenic origins in the gut, we compared and contrasted jReA with other jSpA -Enthesitis-related arthritis (ERA) and undifferentiated SpA (jUSpA).

**Methods:**

A record-based medical data review of jReA, and jUSpA was compared with cohort data of ERA collected for other studies. Data are presented as median (interquartile range) and non-parametric tests used for analysis.

**Results:**

Of 179 juvenile SpA (61 jReA; 101 ERA; and 17 jUSpA), 61 had jReA [M:F-52:9; 15.5 (12–18) years] with a disease duration of 2.75(1–36) months. Inflammatory backache IBP (32%), dactylitis (21%) and enthesitis (29%) were common. A significant proportion (14 of 17, 82.3% at >6 months follow-up) had a chronic course. 101 ERA [M:F-93:7; age-16(14–20) years] had a longer disease duration (45 vs 2.75 months, p<0.001), as compared with jReA. Enthesitis and IBP was more common in ERA (OR-2.3 and 3.4 respectively). jUSpA (n=17) had a similar clinico-laboratory profile and exhibited significant (7 of 17, 58.3%) chronicity over 9.5(4.8–37) months follow-up.

**Conclusion:**

jReA and jSpA exhibit similar features apart from varying disease duration, suggesting that jspA may form a continuum with similar clinico-laboratory profiles plausibly due to shared pathogenesis.

## KEY MESSAGE

jReA and jSpA exhibit similar features suggesting that jspA may form a continuum due to shared pathogenesis.

## INTRODUCTION

Juvenile reactive arthritis (jReA) forms part of the spectrum of spondylarthritis of childhood (jSpA). Despite initial descriptions dating back to the 1960s, the understanding of the disease is limited to a few small case series from literature, the largest one features 44 cases.^1^ The majority of reA reportedly occur between 16 and 35 years, with merely 0.7% of 739 cases described in children below 16 years of age in a large series.^2^ Moreover, there is no data from India, where gut infections and consequently post-infectious rheumatic syndromes are common.

The nomenclature of childhood spondylarthritis is ill-descript, with virtually non-existent data on the clinical spectrum of childhood Undifferentiated spondylarthritis (jUspA). Moreover, comparisons amongst the clinical spectra of childhood SpA have not been drawn previously. Thus, we compared jReA with the various types of childhood spondyloarthropathies, namely ERA (Enthesitis related arthritis) and Undifferentiated spondylarthritis (UspA).

## METHODS

We reviewed hospital records of children following up in the paediatric rheumatology clinic from 1989 to 2017 at a tertiary care centre in northern India. It has been the oldest centre dedicated to rheumatology and clinical immunology research for beyond 30 years. Those with a diagnostic label of jReA (Braun and Seiper’s criteria, 1990),^3^ and Undifferentiated spA (ESSG criteria, 2009)^4^ were included. Data collected for Enthesitis Related Arthritis (ERA, ILAR criteria, 2001)^5^ for previous studies were retrieved for comparison. ERA were further classified into two groups, one which satisfied adult ankylosing spondylitis criteria (ASAS criteria, 2009^6^) named as juvenile ankylosing spondylitis (jAS) and the ones who didn’t fulfil the adult criteria (JIA-ERA). Patients who were 18 years of age or older at the onset of symptoms were excluded.

Baseline patient variables such as demographics, clinical profile (arthritis, tenosynovitis, dactylitis, enthesitis, and other spA features), preceding infections, and the treatment received were gathered. Follow-up outcomes such as persistent pain, new joint or entheseal involvement, axial symptoms, and drug intake were also recorded where available. Comparisons were drawn between jReA and each of the other three groups.

Statistical analysis was done using GraphPad Prism 7 and SPSS version 20. Data are represented as median (Interquartile range). Non-parametric tests (Fisher’s Exact test for proportions and Man-Whitney U test for comparing means) were used. Ethics approval for waiver of consent was taken (Sanjay Gandhi Postgraduate Institute of Medical Sciences ethics committee, Number 2018-124-IMP-105). We followed STROBE checklist for reporting.^7^

## RESULTS

Medical records review from 1989 to 2017 yielded 169 cases of jSpA and 4 mimics. Comparisons drawn between ReA (n=61), UspA (n=17), and ERA (n=101) (**Table 1**).

**Table 1.** Clinical and Laboratory Characteristics of various Juvenile Arthritis.

**Characteristics**	**jReA (n=61)**	**jUspA (n=17)**	**ERA (n=101)**
Age (years, median, IQR)	15.5 (12–18)	17 (16–20)	16 (14–20)*
Disease duration (months)	2.75 (1–36)	12 (3–36)	45 (18–72) ***
Age at disease onset	13 (10.6–15.3)	15 (13–17)	13 (10–15)
Male: Female	52:9 (5.8:1)	14:3(4.7:1)	93:7(13:1)
HLA B27 positivity	15 of 18 (83%)	NA	77 of 83 (93%)

Clinical Profile			
Arthritis	61	17	95
Enthesitis	18	5	50*
Dactylitis	13	-	-
IBP	20	3	63***
Skin lesions	9	1	-
Eye involvement	11	-	14
Uveitis	3	1	-
Conjunctivitis	2	1	-
Keratitis	2$	-	-
Nail changes	-	-	1
Chronic diarrhoea	-	-	-
Other	Chondritis	-	-

Family history of spA	5^&^	1^#^	13

Erythrocyte Sedimentation	26 (55–86)	46 (24.3–85)	8 (3–48)
Rate (mm in 1st hour)			
C- Reactive Protein (mg/dL)	1.8 (4.2–7.6)	2.88 (1.71–3.8)	3 (0–14)

Abbreviations: jReA: Juvenile Reactive Arthritis; jUspA: Juvenile Undifferentiated Spondyloarthritis; ERA: Enthesitis Related Arthritis; spA: Spondyloarthritis.

$ Fungal, keratolysis

& All in the father

# The father has ankylosing spondylitis

^ 17 anytime

* p<0.05

** p<0.005

*** *** p<0.001

### Clinical profile of jReA

**Figure 1.**
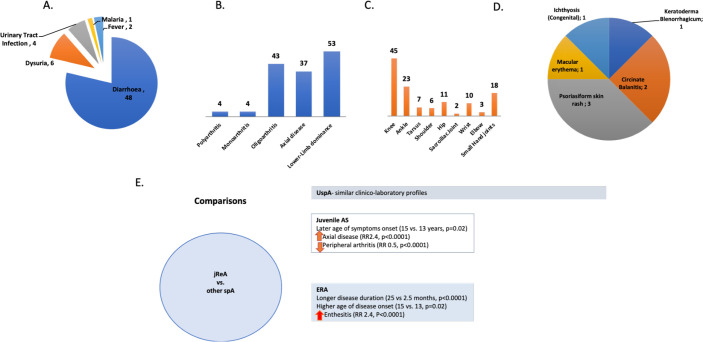
**(A)** Preceding infections. **(B,C)** Joint distribution. **(D)** Extra-articular features. **(E)** Comparison with other juvenile spondyloarthritis.

Post enteric jReA was the most type, seen in 48 (71%) children. The knee joint was most often involved (45, 73%) (**Figure 1**). Other clinical features were inflammatory backache (20, 32%), dactylitis (13, 21%), enthesitis (18, 29%), skin changes (9, 15%), and eye involvement (11, 18%). Five had a positive family history of spA. Of the 17 (27%) children who followed up for ≥6 months, 14 (82.3%) developed chronic/relapsing disease and 12 were still taking drugs. Four of them (23%) eventually were relabelled as ERA, and four (23%) as chronic ReA. The median time to evolution to chronicity and another diagnosis 14 (6–30) months. HLA-B27 positivity was similar as in JIA-ERA (83% vs 93%).

### Clinical profile of jUspA

Assessment of 17 children with undifferentiated spondyloarthritis (ReA like presentation without preceding history of infection) yielded a similar clinico-laboratory profile to jReA. Of the 12 (70%) followed up over 9.5 (4.8–37) months, 7 (58.3%) evolved to chronicity.

### Clinical profile of JIA ERA

Hundred and one children with JIA-ERA of age 16 (14–20) and disease duration 45 (18–72) months were also assessed. Children with ERA had similar gender distribution but longer disease duration (45 vs 2.75 months, p<0.001), and similar age of disease onset as compared with jReA. Enthesitis was more common in ERA [OR 2.3 (1.2–4.6, P=0.011] and also IBP [OR 3.4 (1.7–6.8), p<0.001]. Fourteen (23%) children were reassessed at three months, of which three (21%) were in remission. Rates of remission were similar to those in jReA (p=0.23). Among the subgroup, 41 patients of jAS (who fulfilled ASAS criteria) with age of disease onset 15 (13–16.1) years and disease duration 6.5 (3–12) years were studied. Gender distribution was similar to jReA. Patients with jAS had delayed symptoms onset (15 vs. 13 years, p=0.02) as compared with jReA. Axial symptoms occurred in all with jAS (OR-2.4, p<0.001) while peripheral arthritis was less common (OR-0.5, p<0.001).

Sixty children with JIA-ERA (who didn’t fulfil ASAS criteria) of age 15 (13–16), and disease duration 25 (10.8–48) years were also assessed. Children with ERA had similar gender distribution but longer disease duration (25 vs 2.5 months, p<0.0001), and higher age of disease onset (15 vs. 13, p=0.02) as compared with jReA. Enthesitis was more common in ERA (OR 2.4, P<0.0001). Fourteen (23%) children were reassessed at three months, of which three (21%) were in remission. Rates of remission were similar to those in jReA (p=0.23).

### jReA mimics

During the records review, we found four cases that were initially labelled as jReA but turned out to be mimics. The final diagnoses were post-streptococcal reactive arthritis (PSRA, n=2), disseminated tuberculosis, and anaplastic large cell tumour respectively (**Table 3**). Both the patients with PSRA had upper limb dominant arthritis and had monophasic arthritis. The patient with tuberculosis improved but had severe stunting while the malignancy patient did not survive.

**Table 3.** ReA-like syndromes in children (Clinical Mimics).

**Diagnosis**	**Age (years)**	**Presentation**	**Mode of diagnosis**	**Outcome**
Disseminated Lymphohistiocytic tumour - Anaplastic Large cell lymphoma (LCA+, CD3, ALK-1+,CD30+)	13	Fever- 7 day, dactylitis, knee, elbow and sternoclavicular arthritis→ Later subcutaneous nodules and mandible pain	Nodule Biopsy	Death
Post Streptococcal reA	9	Fever-10 days, wrist, hand joint and ankle arthritis→ later recurrence with rheumatic heart disease	Clinical correlation, Anti-Streptolysin O titre positive	Did well with Penicillin prophylaxis over 4 years
Disseminated Tuberculosis	10	Fever, diarrhoea, Knee arthritis	Red Flags- Growth retardation, monoarthritis Synovial fluid culture positive for AFB	Lost to follow up
Post Streptococcal reA	15	Symmetric upper limb polyarthritis	Elevated Anti-Streptolysin O titers	Subsided after 5 months

Abbreviations: ReA: Reactive Arthritis; AFB: Acid Fast Bacilli

## DISCUSSION

Global research on ReA is limited due to the lower incidence of the disease in economically advanced countries possibly due to improved hygiene and control of infectious diseases.^8,9^ However, Courcoul et al. recently found that in France, the incidence of ReA related to enteric infection was similar across decades.^8^ We found that the various types of jSpA have a fairly similar clinical profile with differences limited to the duration of the disease. JIA-ERA patients were older at presentation and had a longer disease duration. The differences in clinical profile largely paralleled the elements used in the clinical definitions. Thus, enthesitis and IBP were more in ERA. Arthritis was less common in UspA than ReA. Enthesitis was more in ERA. IBP was less common in UspA and more common in ERA. The CRP and ESR levels were similar in the three groups. Notably, the rates of remission were similar across jReA, jUspA, and ERA. Previously Liang et al have described that the clinical profile of childhood ReA and ERA are particularly similar, with minor laboratory differences.^1^ However, their study had primarily focused on laboratory aspects of ReA versus systemic and polyarticular JIA and found a higher ESR and platelets in the acute phase of illness as compared with polyarticular or pauciarticular JIA (**Table 2**).

**Table 2.** Comparison with other cohorts.

**Characteristics**	**jReA (n=61) Our Study**	**jReA (n=44) Liang et al.**	**jReA (n=39) Stefanov et al.**	**jReA (n=26) Cuttica et al.**
Disease duration (months)	2.75 (1–36)	NA	NA	28.6
Age at disease onset	13 (10.6–15.3)	5.8 (0.6–14.3)	10.8 (3–17)	10.5 (4–15.5)
Male: Female	52:9 (5.8:1)	30:14 (2.1:1)	26:13 (2:1) ^*^	22:4 (5.5:1)
HLA B 27 Positivity	15 out of 18 (83%)	NA	21 of 32 (65.6%)	12 of 18 (67%)
Chronic disease (>6 months)	14 out of 17 (82 %)	NA	NA	10 (38.5 %) ^**^

Clinical Profile				
Arthritis	61 (100 %)	NA	39 (100 %)	26 (100%)
Enthesitis	18 (29 %)		-	-
Dactylitis	13 (21.3 %)		3 (7.7 %)	-
IBP	20 (32.7 %)		9 (23%)	5 (21%)
Skin lesions	9 (14.7 %)		7 (17.9 %)	11 (50%)
Eye involvement	7 (11.4 %)		29 (74.4 %) ^***^	-
Uveitis	3 (4.9 %)		-	-
Conjunctivitis	2 (3.2 %)		29 (74.4 %) ^***^	-
Keratitis	2$ (3.2 %)		-	-
Nail changes	-		-	-
Chronic Diarrhoea	-		-	-
Other	Chondritis		-	-

Family history of spA	5	NA	NA	NA

Erythrocyte Sedimentation Rate (mm in 1st hour)	26 (55–86)	37 (5–108)	NA	NA
C- Reactive Protein (mg/dL)	1.8 (4.2–7.6)	2.1 (0–9.8)	NA	NA

Abbreviations: jReA: Juvenile Reactive Arthritis; spA: Spondyloarthritis.

* p<0.05

** p<0.005

*** p<0.001 (Chi square)

The pathogenesis of reA involves an infection trigger Th2 mediated immune response. Cross reaction and molecular mimicry may be possible mechanisms. HLA-B27 is positive in 60%–70% of ReA mediating its spondylarthritis symptoms. Further, studies have shown that activating killer cell immunoglobulin like receptor KIR2DS1, is associated with susceptibility to ReA when present in association with HLA-C ligand genotypes.^10^ Bacterial DNA of Chlamydia may be found in synovial fluid. Tetracycline antibiotics have shown to reduce the duration of chlamydia related ReA and not enteric related ReA.^11^ Treatment with antibiotics doxycycline plus rifampicin was superior to doxycycline alone in reducing tender and swollen joints in reA.^12^

The Greek cohort of juvenile ReA patients had some peculiar findings like pericarditis in 2 (of 9) patients which were not found in other cohorts.^13^ Otherwise, the age of onset, disease duration and HLA-B27 associations were similar. While diarrhoea was the most common preceding infection in this study, in Taiwan, the upper respiratory infection was the leading cause, being implicated in 52.3% of children.^1^ Diarrhoea occurred in eight of these children, amounting to 18.1% of the cases. A plethora of atypical preceding illnesses such as herpangina, hand foot and mouth disease, infectious mononucleosis, chicken pox, and aseptic meningitis due to enteroviruses were also reported. This suggests the widened spectrum of preceding infections and the varied nature of such infections in the developed versus the developing world. Since the first descriptions of ReA, to describe a post-Yersinia non-infective arthritis,^14^ the spectrum has significantly broadened. With the changing profile of infections in the era of HIV infection and the widespread use of potent immunosuppressants such as biologics for rheumatic and other autoimmune and allergic diseases, the spectrum of infections has also evolved greatly.^15,16^

JReA, albeit more common in males in both cohorts, had a lower predilection for boys in the Taiwanese cohort. This could be a function of the different prevalence of HLA B27^17^ or even gender imbalances in access to clinical care in India. Comparing two other studies of 39 and 26 patients each, the HLA rates are reflective of those expected in the Indian population, though higher chronicity could arise out of a self-selection follow-up bias (**Table 2**). Further conjunctivitis was seen in lower proportions when compared to Stefanov et al.^18^ and chronicity was more than that seen in Cuttica et al.^19^ (**Table 2**). In comparison to Liang et al. and Stefanov et al., our cohort of jReA patients had more male preponderance, whereas it was similar to Cuttica et al. cohort. Additionally, monoarthritis was much infrequent in the Indian cohort as compared with the Taiwanese.^1^ This could be due to earlier referral and prompt medical care as seen from the much shorter disease duration in the Taiwanese cohort. This also reiterates the additive nature of arthritis in ReA.

We could not find published data on UspA in children. However, the glaring similarity in clinical features with ReA highlights that these could very well be cases with the latter without a molecular diagnosis or a recall bias in the history of preceding infection. In such cases, looking for circinate balanitis or keratoderma can yield the correct diagnosis. This highlights the need for a more realistic case definition of ReA. The diagnostic criteria for ReA (3) the relevance seems to be only that antigenic material reaches the joint, alive or dead. If there is a common antigen, it has to be a highly conserved one. Bacterial hsp60 seems to be an immunodominant T cell antigen in ReA, but there must be other relevant antigens shared by these different bacteria. An ineffective immune response (for example, low production of TNFalpha, which require evidence of preceding microbial infection as determined by culture, DNA, RNA, or antibodies to the infecting agent, are too rigid to be practical. Thus, most patients are at most diagnosed at probable reA.^20^ Moreover, serologic evidence might be impractical due to the high background immunity in the developing world.

It is worth mentioning that many children with Enthesitis-related arthritis can very well fit into the criteria for the diagnosis of chronic ReA. Gut infections have been observed to induce flares of ERA in children.^13,21^ The International League Against Rheumatism classification scheme for JIA does not mention jReA as a subtype.^5^ This suggests the need to revise the diagnostic criteria for JIA to be more inclusive of all forms in children. HLA B27 positive childhood arthritis is clinically similar to AS and ReA but generally does not fulfill criteria.^22^ Synovial fluid mononuclear cells from ReA, UspA, and ERA exhibit similar lymphoproliferation and cytokine production in response to gut bacterial antigens such as S Typhimurium.^21,23^. In light of the above, it seems plausible that ReA and other SpA form a continuum with a shared pathogenic basis.^8^

Lastly, the diagnosis of tuberculosis and anaplastic large cell tumor in two children presenting with ReA like syndromes emphasizes to keep a high index of suspicion for atypical features such as upper limb arthritis, prolonged fever, subcutaneous nodules or mandible pain as has been described in previous studies.^24^

This is the largest series describing the clinical profile of jReA to date. It has the unique advantage of comparisons drawn between the various types of childhood spondyloarthropathies and jreA mimics. It offers a glimpse into the possibility of a common origin with the blurred distinction drawn merely by semantics. The main limitations are those inherent to a medical record review-based data set, resulting in a high attrition rate which may be mitigated with a planned prospective cohort with active follow-up. Further, our study predates the Martini et al.^25^ classification of JIA and the retrospective data may not do justice to the current definitions. Moreover, data in jreA and jUspA was record-based while ERA was interview-based, bringing in potential for bias. However, we hope the unique insights offered by this very first study exploring this area with a large sample size of different childhood spA may be confirmed in a global multicentre prospective study.

## CONCLUSION

JReA and jSpA exhibit similar features apart from varying disease duration, suggesting that jSpA may form a continuum with similar clinico-laboratory profiles. This suggests a possibility of shared pathogenesis which merits further investigation. Since these groups have relatively small numbers, it may make sense to club them together while studying pathogenesis and management.
